# Supplementary material for: Structure and mechanism of a tripartite ATP-independent periplasmic TRAP transporter
Source: Nat Commun. 2023 Feb 27;14:1120. doi: 10.1038/s41467-023-36590-1 (PMC9971032; doi:10.1038/s41467-023-36590-1)
Supplement: Supplementary file 1 — Supplementary Information [file 41467_2023_36590_MOESM1_ESM.pdf]

Supplementary Information for:

Structure and mechanism of a tripartite ATP-independent periplasmic TRAP transporter

James S. Davies, Michael J. Currie, Rachel A. North, Mariafrancesca Scalise, Joshua D. Wright, Jack M. Copping, Daniela M. Remus, Ashutosh Gulati, Dustin R. Morado, Sam A. Jamieson, Michael C. Newton-Vesty, Gayan S. Abeysekera, Subramanian Ramaswamy, Rosmarie Friemann, Soichi Wakatsuki, Jane R. Allison, Cesare Indiveri, David Drew, Peter D. Mace, Renwick C.J. Dobson

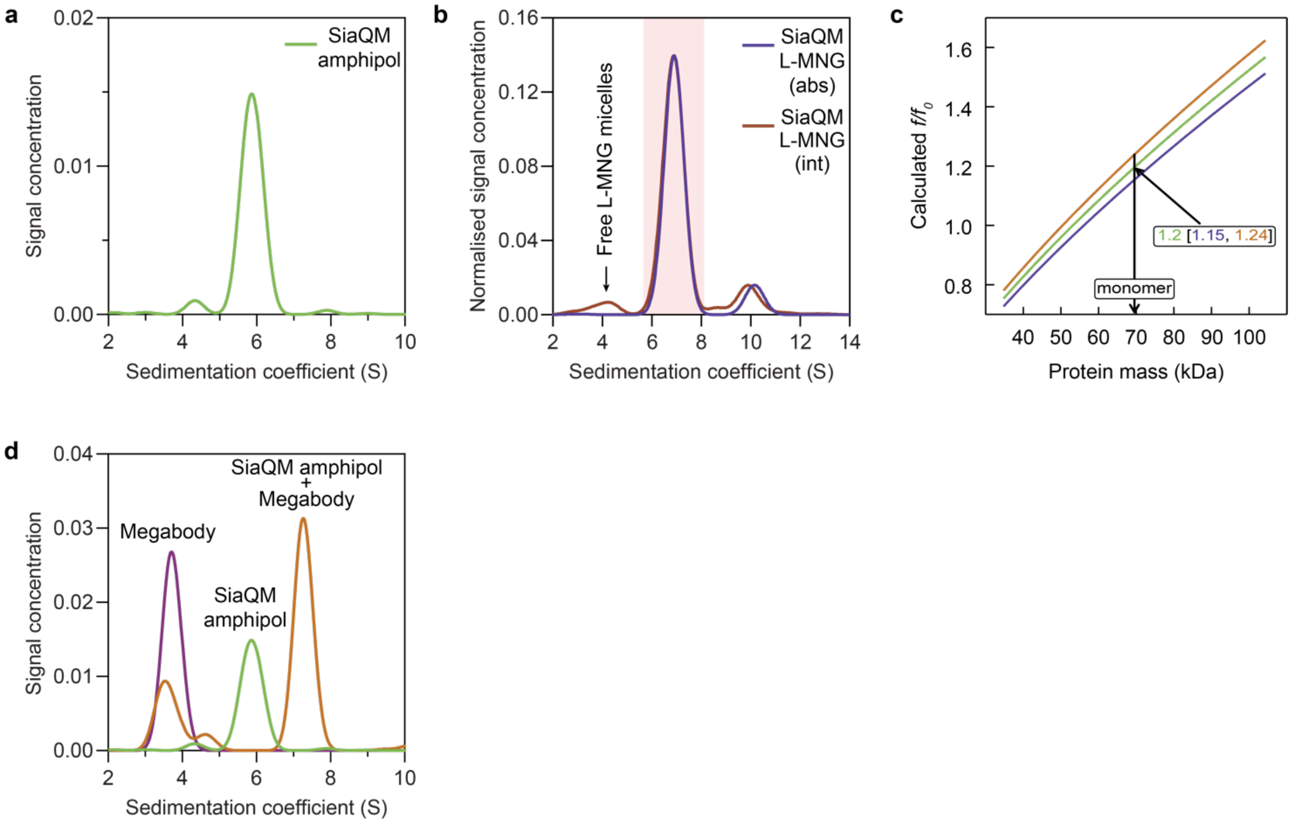

Supplementary Fig. 1 | The interactions between SiaQM: SiaP and $\text{Mb}^{\text{c7HopQ}}_{\text{Nb07}}$, and the SiaQ: SiaM complex stoichiometry. **a**, Sedimentation velocity data for SiaQM in amphipol demonstrates a dominant peak at a sedimentation coefficient of ~5.9 S, consistent with a single species in solution. **b**, Sedimentation velocity data for SiaQM in L-MNG (0.002%) shows a dominant monomeric species. Data collected using absorbance optics is shown in blue and that collected simultaneously using interferometry is shown in brown. The peak at ~4 S seen in the interferometry experiment represents free L-MNG micelles. A small proportion appears to exist as a dimer of heterodimers (~10 S)—which may be due to micelle cohabitation. **c**, Sedimentation data analysis[1] demonstrates that the major species at 6.5 S in **b** (shaded) is most consistent with a monomeric transport unit of SiaQM (that is, one SiaQ and one SiaM form the complex). After determining the amount of L-MNG bound to the protein with laser interferometry, the calculated f/f_0 for a monomer (heterodimer) for the major species in **b** is 1.2 (1σ error = 1.15−1.24), consistent with a protein in a detergent micelle. The

calculated f/f_0 for a dimer (heterotetramer) for the major species in **b** is 1.9 (1σ error = 1.83−1.97) (not shown), which is much less likely. The calculated mass of the protein from **b** was 68.2 kDa, consistent with a monomeric mass of 69.5 kDa determined from the protein sequence. The protein-detergent complex was calculated to have 83 ± 4 molecules of L-MNG bound for a total mass of the sedimenting complex of ~152 kDa. **d**, Sedimentation velocity data for SiaQM in amphipol (green), Mb_{Nb07}^{c7HopQ} (purple) and combined (1:1.5 ratio, orange) shows a distinct increase in sedimentation coefficient for the bound species (orange). The peak at ~3.5 S (orange trace) is excess unbound megabody. Source data are provided as a Source Data file.

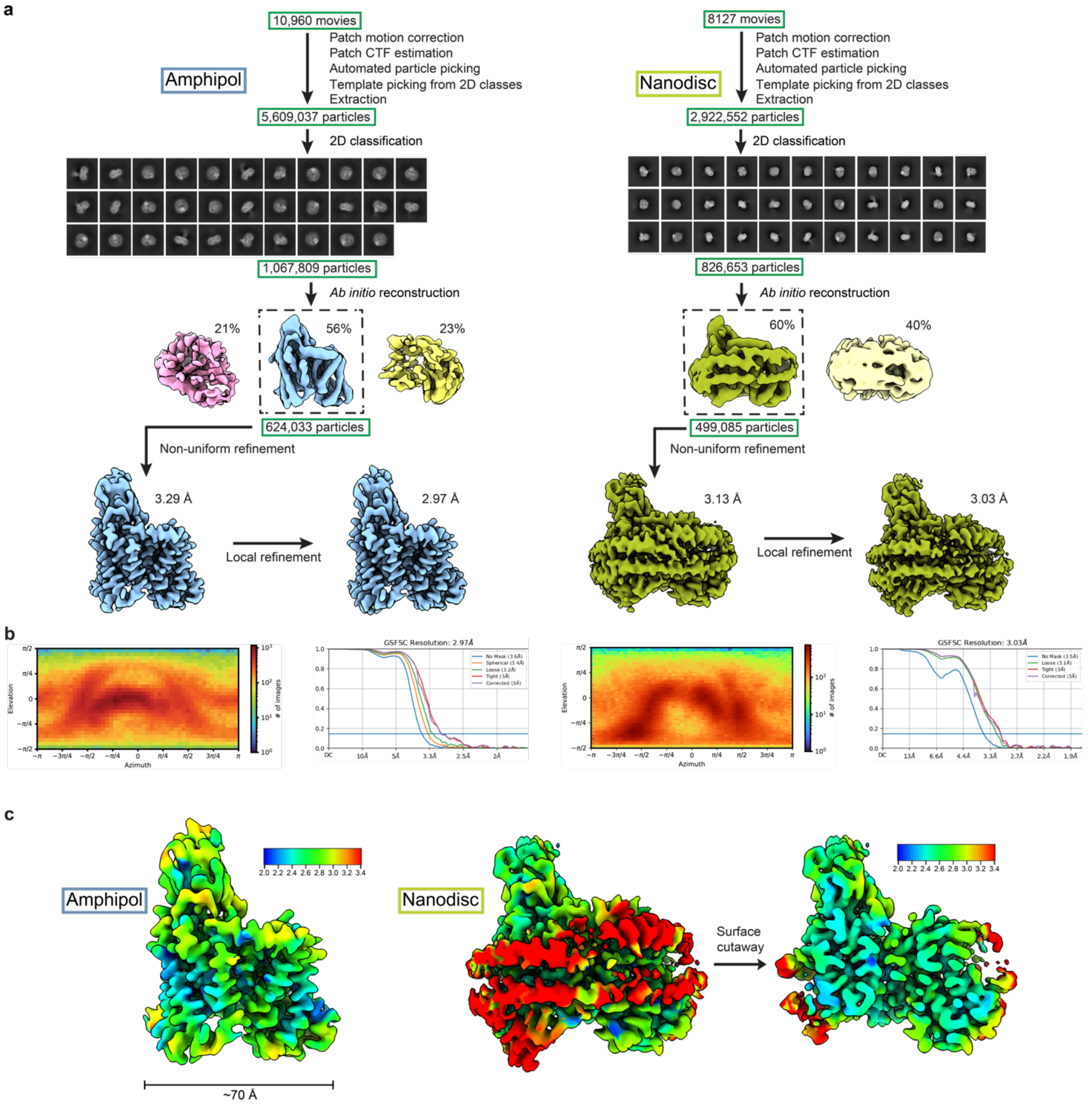

Supplementary Fig. 2 | Cryo-EM workflow and analysis of the SiaQM-Mb$^{\mathrm{c7HopQ}}_{\mathrm{Nb07}}$ complex. a, Workflow outlining the cryo-EM image acquisition and processing to obtain a structure of SiaQM-Mb$^{\mathrm{c7HopQ}}_{\mathrm{Nb07}}$ solubilised in amphipol (left) and nanodiscs (right). The selected 2D class averages used for *ab initio* reconstruction are shown. The *ab initio* reconstructions were separated into multiple classes to remove junk particles and the best 3D reconstructions were used as reference models for non-uniform refinement. Masks were made in RELION 3.0[2] and the maps were further refined with iterative rounds of local refinement. **b**, Viewing direction distribution plot and Fourier shell correlation (FSC) curves for the final 3D reconstructions of SiaQM-Mb$^{\mathrm{c7HopQ}}_{\mathrm{Nb07}}$. **c**, Local resolution maps of SiaQM-Mb$^{\mathrm{c7HopQ}}_{\mathrm{Nb07}}$ showing that the data extends to 2.2 Å in some areas.

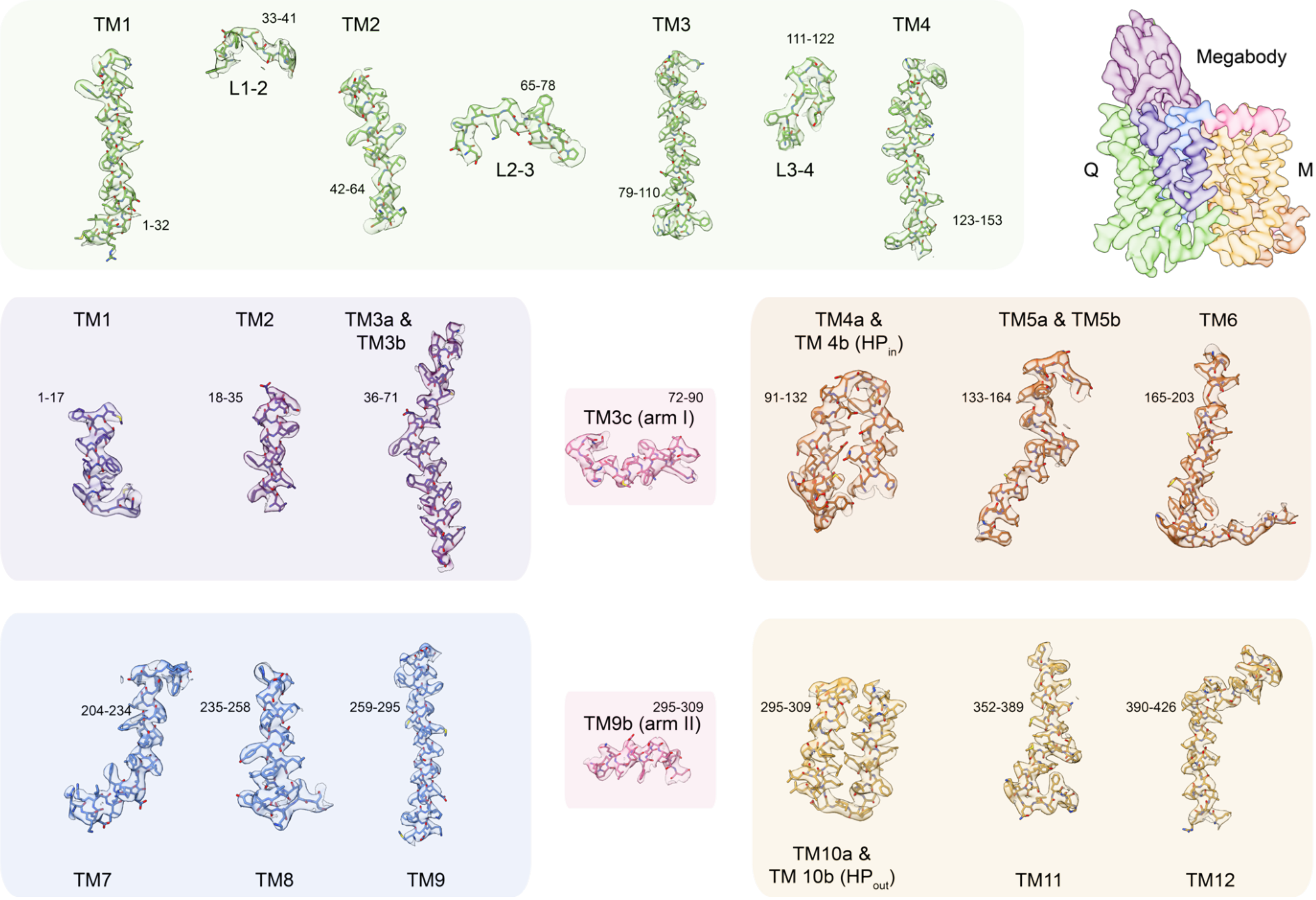

Supplementary Fig. 3 | Cryo-EM density of the helices of SiaQ and SiaM. The amphipol map level is set at 7.5 σ, as calculated by *ChimeraX*[3].

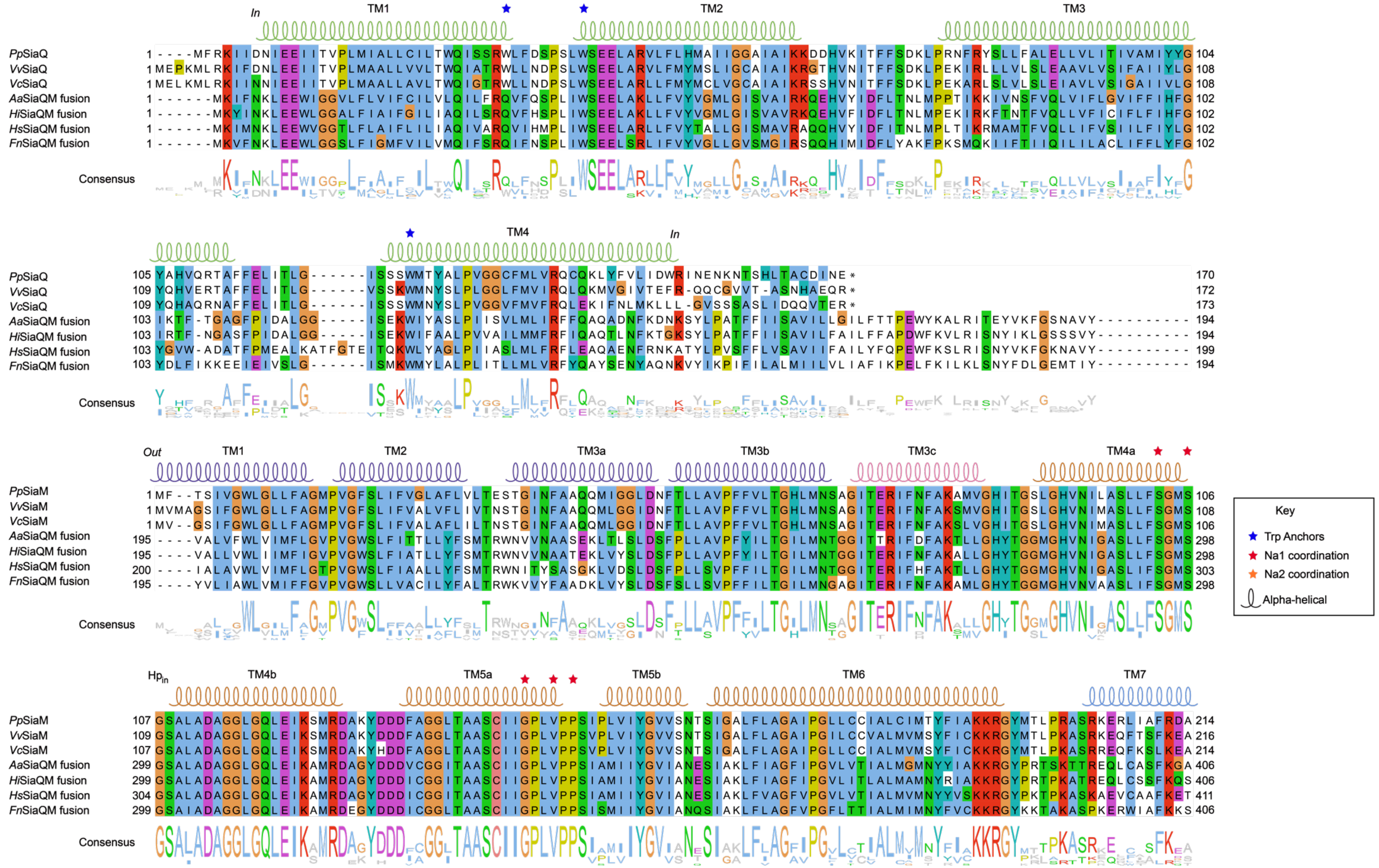

Supplementary Fig. 4 | SiaQM protein sequence alignment (continued on next page)

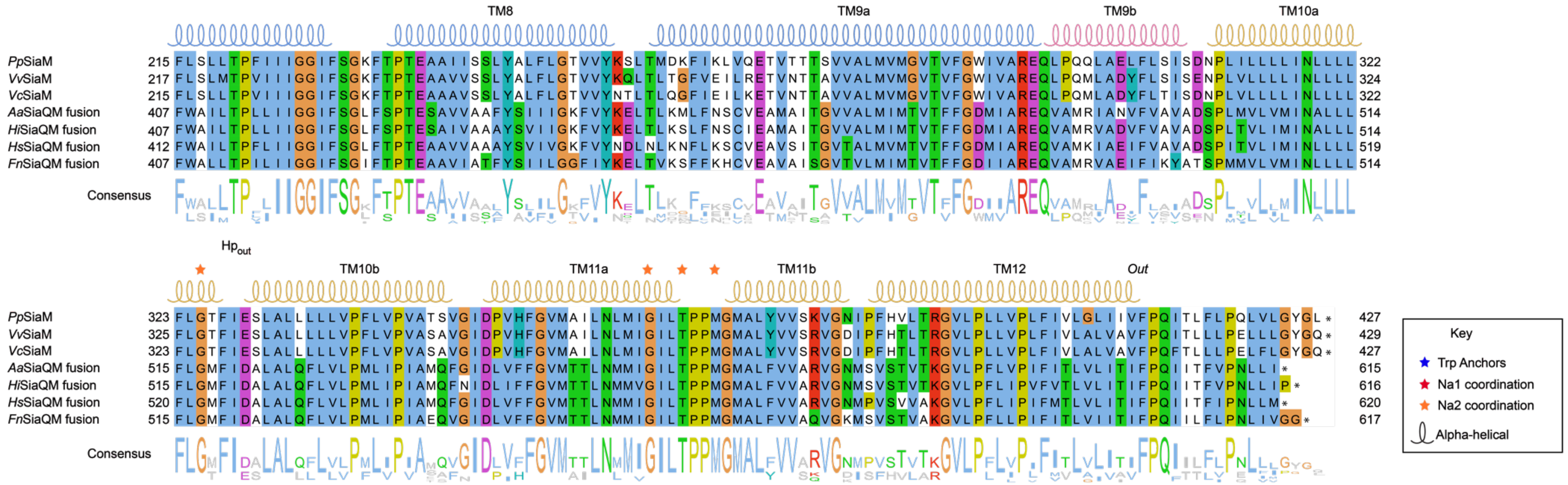

Supplementary Fig. 4 | SiaQM protein sequence alignment. SiaQM protein sequences from *Photobacterium profundum (Pp), Vibrio vulnificus (Vv), Vibrio cholerae (Vc), Aggregatibacter actinomycetemcomitans (Aa), Haemophilus influenzae (Hi), Histophilus somni (Hs)* and *Fusobacterium nucleatum (Fn)*. Protein sequences were aligned using AlignMe[4], manually adjusted where appropriate, and coloured in *Jalview*[5] with Clustal X colouring. Blue stars indicate anchoring tryptophan residues, red stars indicate Na1 coordinating residues and orange stars indicate Na2 coordinating residues.

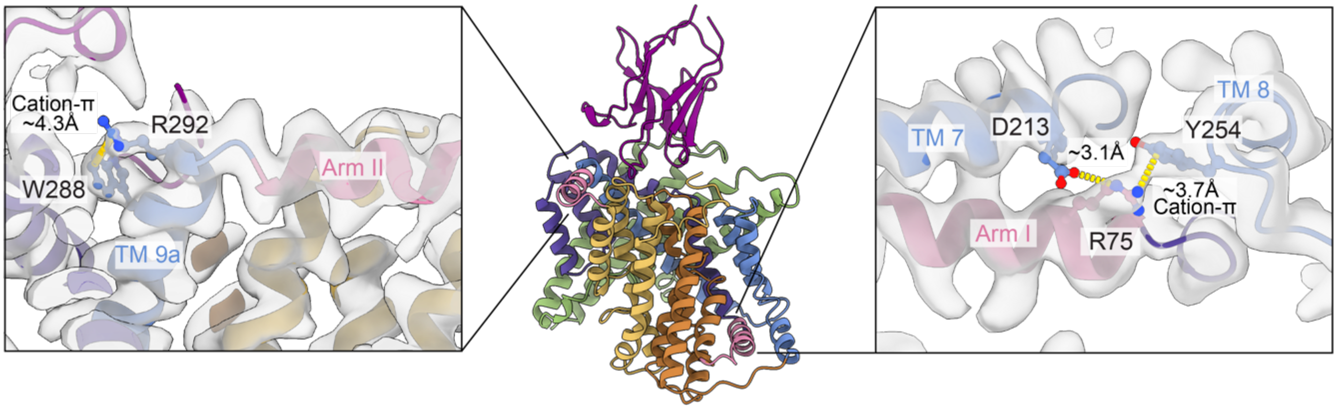

Supplementary Fig. 5 | Interactions between the arm and scaffold helices. Left, inset, a cation-π interaction (W288–R292) is evident at the junction of TM9a (scaffold II) and the amphipathic arm helix II. Right, inset, the highly conserved R75 has a cation-π interaction with Y254 on TM8 (scaffold II), and a potential electrostatic interaction with D213 on TM7 (scaffold II). Cryo-EM density is shown in grey, contoured at 7.5σ. These bulky interactions at the "elbows" of the arm helices are akin to those recently described in DASS transporters[6].

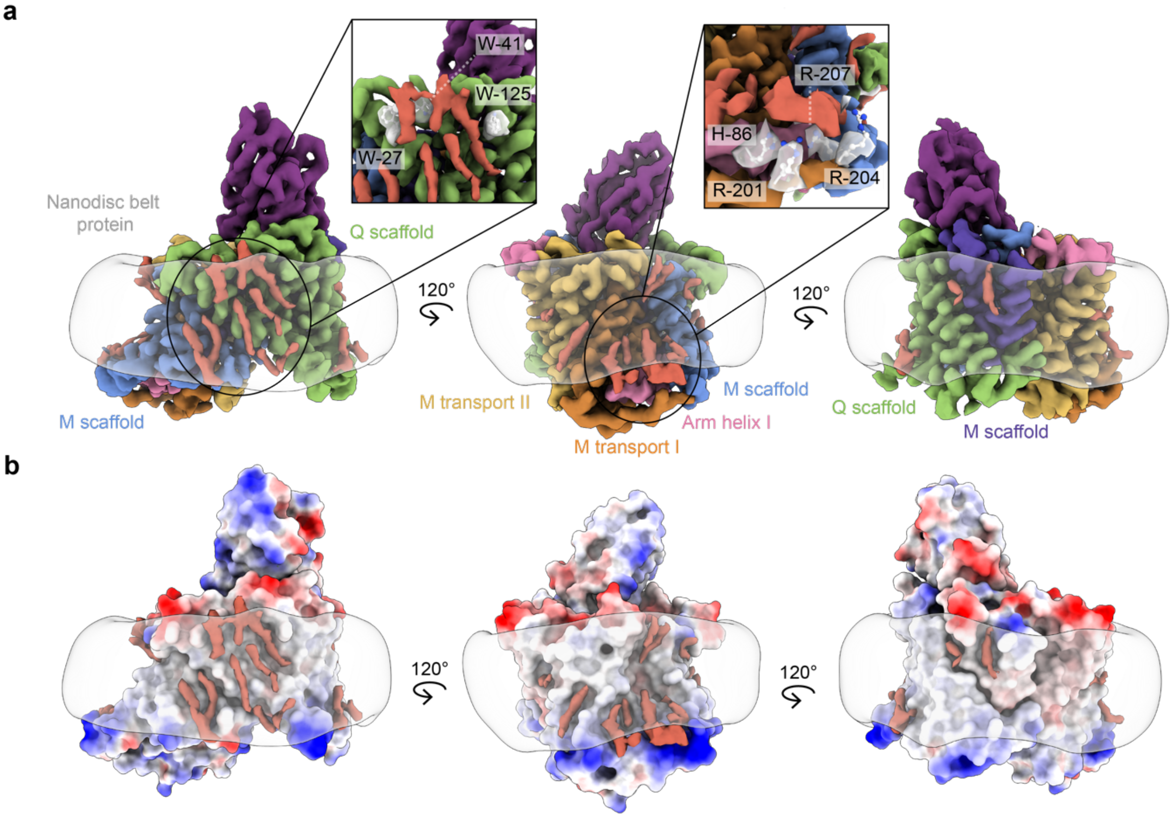

Supplementary Fig. 6 | Lipid densities observed in SiaQM-Mb$^{c7HopQ}_{Nb07}$ nanodisc map. a, Cryo-EM density of SiaQM contoured at 8.3σ and coloured according to domains. Densities corresponding to lipid molecules are coloured in pink. Distinct densities can be seen adhering to the Q scaffold, adjacent to a number of tryptophan residues (left inset). One full lipid (phosphatidylethanolamine) was modelled in the density near W125, with alkanes fitted to the rest of the lipid-like densities. We also note a second prominent site with a number of lipid-like densities, near Arm Helix I (middle inset). At this site is a cluster of positively charged residues near the position of the lipid headgroup, supporting that a lipid with anionic character may interact here. In **b,** the surface of the fitted model is coloured by electrostatic potential, highlighting differences in charge between sites.

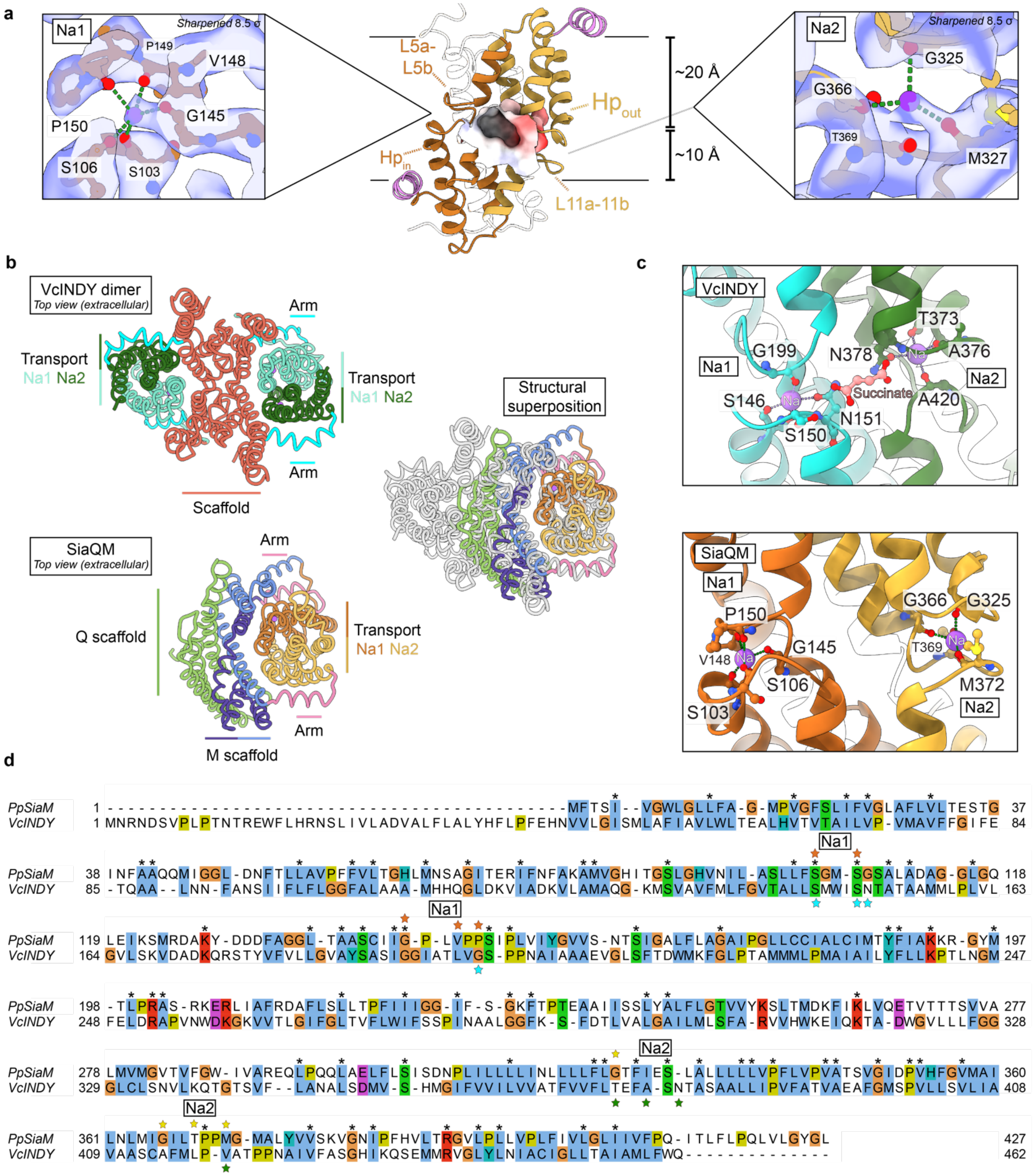

Supplementary Fig. 7 | Cutaway showing the substrate-binding cavity and sodium ion sites. a, The depth of the substrate-binding site is indicated, as estimated from membrane thickness calculations[7]. The cavity that forms the sialic acid binding site (*middle*) is coloured by electrostatic surface and flanked by the sodium ion sites, Na1 and Na2. The Na1 site is shown inset (*left*), with the cryo-EM density in blue, contoured at 8.5σ from an automatically sharpened map generated by *PHENIX*[8]. The Na2 site is shown

inset (*right*), with likely coordinating residues indicated. At both sites are conserved twin proline motifs, modelled here in the *cis* conformation (supported by *AlphaFold* predictions). **b**, A structural overlay of the VcINDY and SiaQM transport domains. **c**, The assignment of the sodium ion sites (*left*) with likely coordinating residues is supported by structural comparison with VcINDY (PDB ID: 5ul7) (*middle, right*). **d,** Sequence alignment of SiaM and VcINDY. Protein sequences were aligned using AlignMe[4] and coloured in *Jalview*[5] with Clustal X colouring. Asterisks indicate conserved residues, sequence identity = 15%. Stars indicate Na^+ coordinating residues in each sequence.

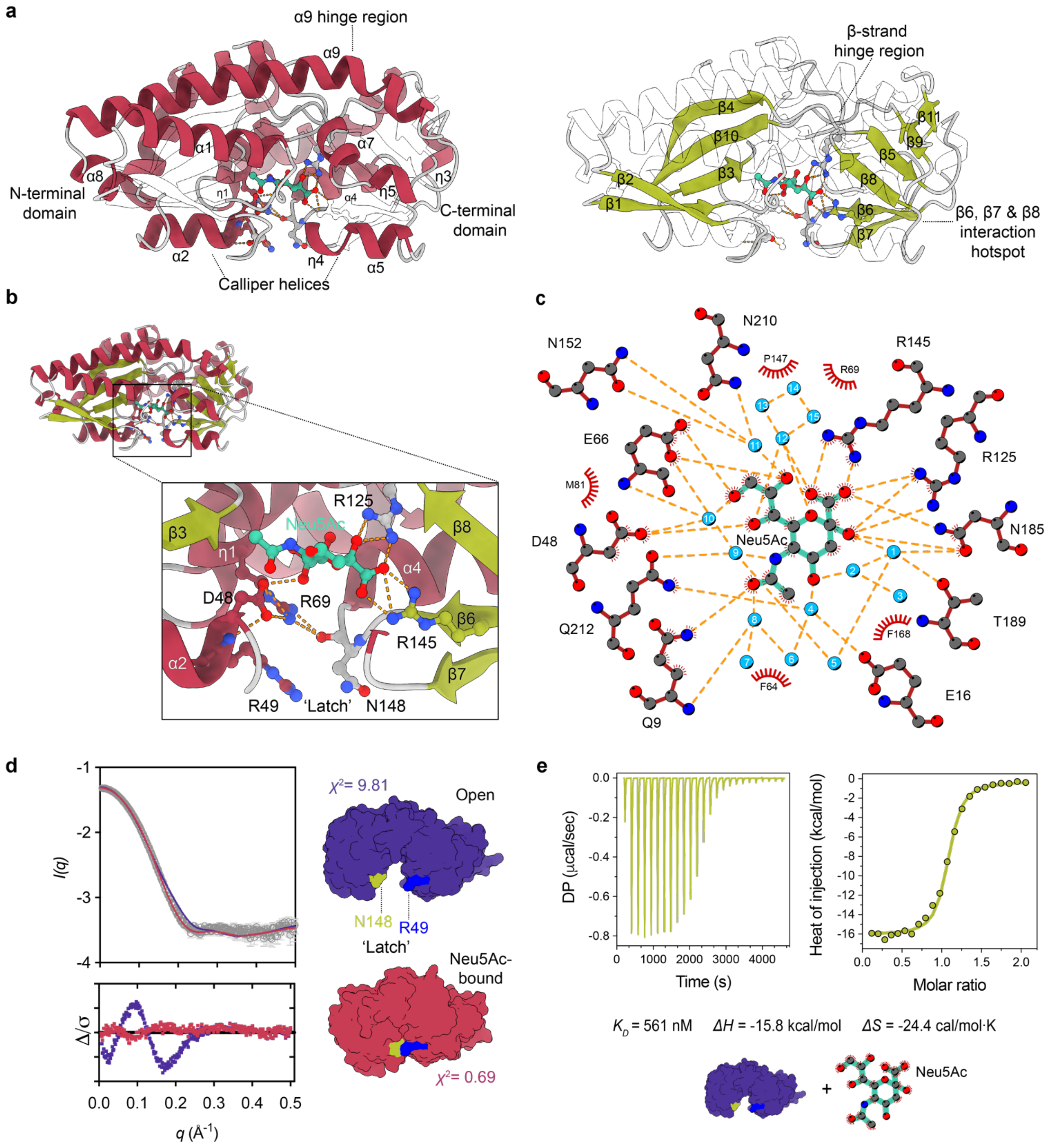

Supplementary Fig. 8 | Structure of SiaP. **a,** Structural features of SiaP, including the 'mixed-hinge' comprised of a kinked helix α9 and two twisted β-strands (one comprised β4 and β5, and the other β9 and β10). **b,** Hydrogen bonding networks from a surface 'latch' area to Neu5Ac (cyan) in the binding cleft. D48 appears to play a central role, interacting with residues from both N- and C-terminal domains, and also with the hydroxyl of C7 in Neu5Ac, and a highly ordered water (10, in Ligplot). **c,** Ligplot[9] showing close interactions at the SiaP substrate-binding site. Waters are arbitrarily numbered 1–15. **d,** SiaP small angle X-ray scattering data, showing that the X-ray crystal structure of SiaP-Neu5Ac closely

resembles the conformations of bound SiaP in solution. The SAXS data were collected on a single protein sample, with and without the presence of Neu5Ac. In purple is a homology model of SiaP in an open state generated with *Modeller*[10], using PDB ID: 4mag as a template, and in maroon, the SiaP closed structure reported here. In green and blue are 'latch' residues, illustrating their proximity in the closed structure. **e,** Isothermal titration calorimetry (ITC) data shows that Neu5Ac binds to SiaP with nanomolar affinity. Shown is one of three replicate experiments. Global fitting of three replicate experiments using *Sedphat*[11] gives a K_D of 561 nM (68% confidence interval = 446 – 876 nM), ΔH of -15.8 kcal/mol (68% confidence interval = -15.2 – -16.9 kcal/mol), and ΔS of -24.4 cal/mol.K (68% confidence interval = -21.9 – -27.6 cal/mol.K). Source data are provided as a Source Data file.

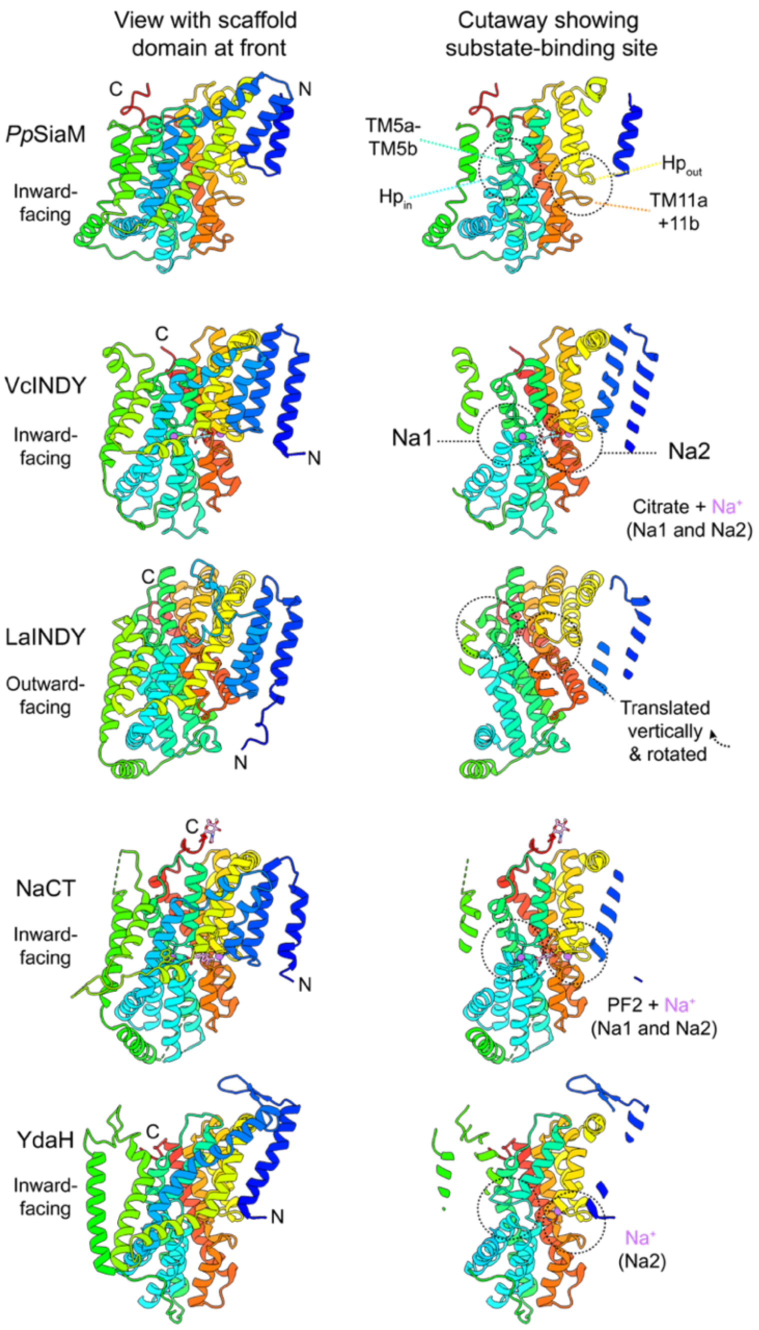

Supplementary Fig. 9 | Comparison of the M-subunit fold with other elevator transporters. (*Left*) Elevator transporters oriented with the scaffold region facing the reader. (*Right*) Cutaway of the same view revealing the transport domain. The two 'clam-shell' motifs[12] of the transport domain are shown in dashed circles, and contribute to the substrate/Na^+ binding site in each transporter. Each clam-shell motif is formed at the interface of a helical hairpin and the break in a discontinuous helix. In SiaM, the two clam-shells are formed by Hp_{out} and the unwound region of TM11, as well as Hp_{in}, and the unwound region of TM5. VcINDY (PDB ID: 5ul9)[12] and LaINDY (PDB ID: 6wu1)[6] are both members of the DASS family, and belong to the wider Ion Transporter (IT) superfamily. As illustrated, LaINDY is the only structure of this fold in an outward-facing state, clearly showing an elevator-type movement in comparison with the homologous VcINDY. YdaH (PDB ID: 4r0c) is a member of the AbgT family[13],

and is also a member of the wider IT superfamily. A distinguishing feature of the M-subunit of SiaQM is its topology—the other transporters displayed here all have their N-terminal helix (blue), inserted from the cytoplasm. The M-subunit shares a conserved fold with VcINDY (4.8 Å r.m.s.d. over 352 residues, PDB ID: 5ul7) and NaCT (4.2 Å r.m.s.d. over 192 residues, PDB ID: 7jsj), as well as the bacterial AbgT-type transporter, YdaH (4.7 Å r.m.s.d. over 216 residues, PDB ID: 4r0c), which are all members of the Ion Transporter (IT) superfamily[14]. Furthermore, these transporters form homodimers at their scaffold domains (blue, yellow and green), which is where the Q-subunit binds. The displayed transporters also show other variations such as loop length, sequence insertions, and the angle and position of TMs.

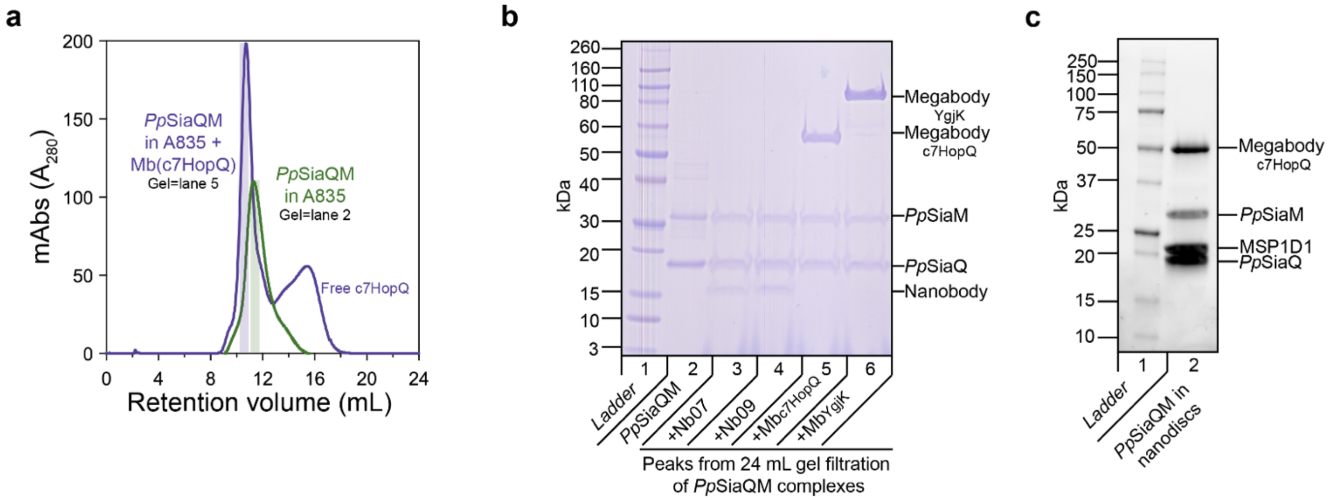

Supplementary Fig. 10 | Purification of SiaQM-Mb$^{c7HopQ}_{Nb07}$ for single particle analysis. **a,** Size-exclusion of SiaQM solubilised in A8-35 (green) and SiaQM-Mb$^{c7HopQ}_{Nb07}$ complex (purple). **b,** SDS-PAGE of peaks from size-exclusion. Lane 2 corresponds to the middle of the green peak, while Lane 5 corresponds to the middle of the purple peak, as indicated. SiaM (~45.4 kDa) does not run true to size on SDS-PAGE, which has been documented previously[15]. An additional nanobody (Nb09, Lane 4) and megabody (MbYgjK, Lane 6) were screened but not used in this study. This experiment was conducted once. **c,** SDS-PAGE of SiaQM reconstituted into MSP1D1-*E. coli* phospholipid nanodiscs. This experiment was conducted once. Lane 2 shows the sample used for cryo-EM. Source data are provided as a Source Data file.

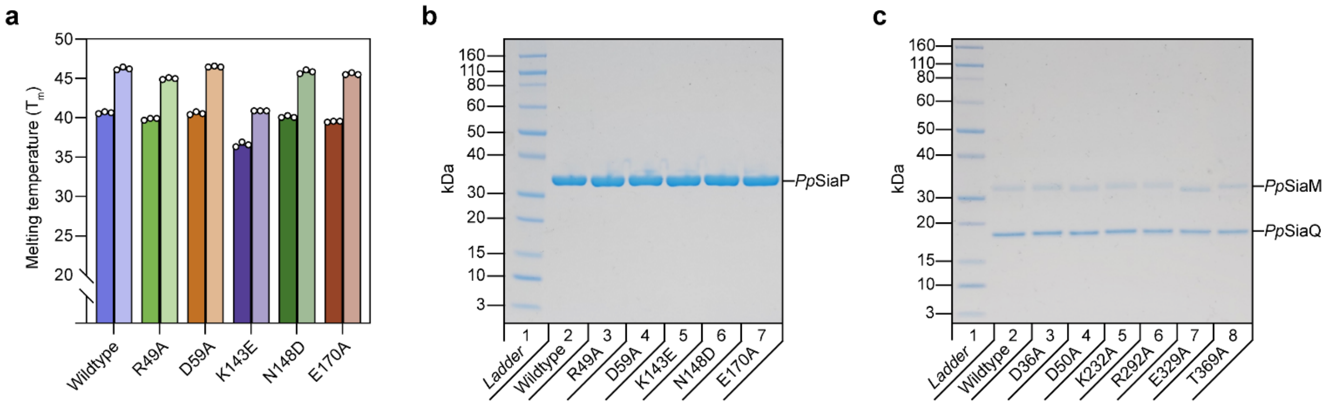

Supplementary Fig. 11 | Temperature stability, sialic acid binding and purity of SiaP and SiaQM mutants. a, A thermal shift assay was used to determine the ability of the SiaP mutants to bind sialic acid. Each of the mutant proteins increased in melting temperature by 4.3-6.0 °C in the presence of sialic acid (lighter colour of each pair), replicating the increase seen in wildtype SiaP. Bars are plotted as the mean of three replicates (white circles). **b,** SDS-PAGE of purified SiaP mutants shows that the purity is the same as the wildtype. The SiaP mutants D181A, D181K, F195-E197A were unstable and could not be isolated. **c**, SDS-PAGE of purified SiaQM mutants shows that the purity is the same as the wildtype. Source data are provided as a Source Data file.

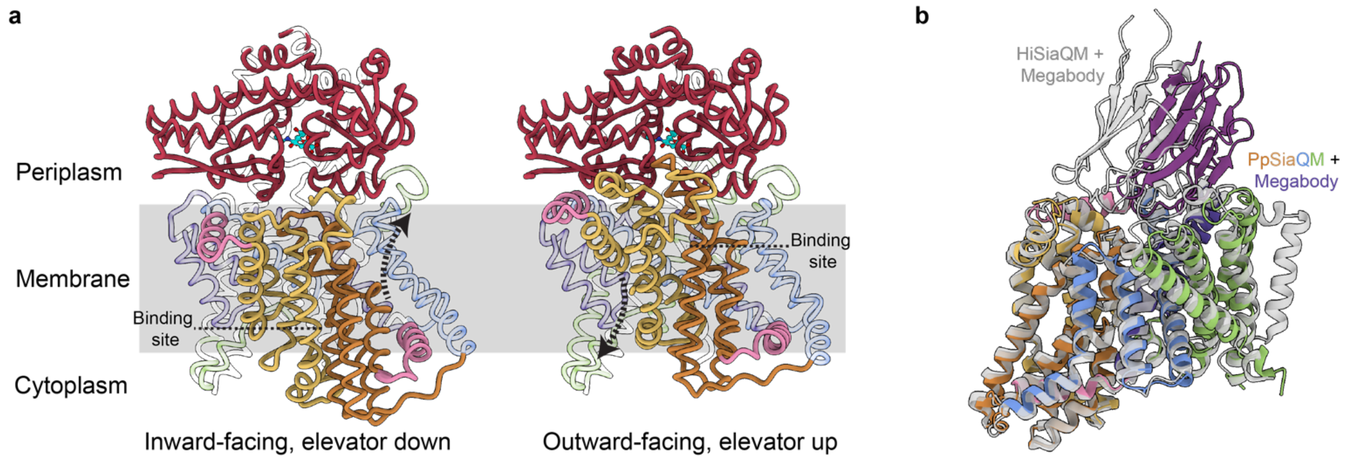

Supplementary Fig. 12 | a, Modelling of the elevator motion of *Pp*SiaPQM. *Left* Tripartite model using experimental structures. *Right* Tripartite model using homology model based on LaINDY structure. The transport domain (coloured orange and yellow) shows a vertical translation and rotation, as indicated by the arrows. **b**, *Pp*SiaQM structural alignment to *Hi*SiaQM (PDB ID: 7qe5). The RMSD between all 424 Cα pairs calculated by *ChimeraX* was 1.439 Å. The alignment shows the two structures adopt the same inward-facing downward conformation, with subtle differences observed in the position and structure of the loop regions.

Supplementary Table 1 | Cryo-EM data collection, refinement and validation statistics.

	SiaQM-Mb^{c7HopQ}_{Nb07} (amphipol)	SiaQM-Mb^{c7HopQ}_{Nb07} (nanodisc)
**Data collection and processing**		
Magnification	130,000	130,000
Voltage (kV)	300	300
Electron exposure ($e^{-}/Å^{2}$)	71.3	70.9
Defocus range (μm)	-2.0 to -0.4	-2.0 to -0.4
Pixel size (Å)	0.6645	0.6645
Symmetry imposed	C1	C1
Initial particle images (no.)	5,609,037	2,922,552
Final particle images (no.)	624,033	499,085
Map resolution (Å)	2.97	3.03
FSC threshold	0.143	0.143
Map resolution range (Å)	2.2 to 3.3	2.2 to 3.3
**Refinement**		
Initial model used (PDB code)	n/a	n/a
Model resolution (Å)	2.97	3.03
FSC threshold	0.143	0.143
Model resolution range (Å)	2.86−3.31	2.88-3.13
Model composition		
Non-hydrogen atoms	5280	5478
Protein residues	684	684
B-factors ($Å^{2}$)		
Protein (mean)	143.3	124.0
R.m.s. deviations		
Bond lengths (Å)	0.003	0.005
Bond angles (°)	0.595	0.992
Validation		
MolProbity score	1.49	1.25
Clashscore	6.68	2.91
CaBLAM outliers (%)	1.04	1.19
Rotamer outliers (%)	0	0.53
EMRinger score	2.37	2.94
Ramachandran plot		
Favoured (%)	97.35	97.05
Allowed (%)	2.65	2.95
Disallowed (%)	0	0
PDB	7qha	8b01
EMDB	EMD-13968	EMD-15775

Supplementary Table 2 | Enrichment of tryptophan residues in TRAP transporter sequences.

TRAP transporter system	Q gene	M gene	% Trp of scaffold domain	% Trp of transport domain
1	PBPRA_2280	PBPRA_2279	1.9	0
2	VC_1778	VC_1777	1.6	0
3	VVA_1201	VVA_1200	1.6	0
4	Bbta_0129	Bbta_0130	1.1	0.9
5	Xaut_3367	Xaut_3366	2.3	1.3
6	Bpro_1870	Bpro_1869	3.3	0.4
7	Veis_3955	Veis_3956	2.7	0.9
8	PA_5168	PA_5169	2.2	1.3
9	RPB_3328	RPB_3327	1.6	0.9
10	Csal_0661	Csal_0662	2.2	0.9
11	Dde_1549	Dde_1550	1.6	0.9
12	Desal_0341	Desal_0340	1.6	0.9
13	FN_1257	FN_1256	0.9	0.4
14	Asuc_0157	Asuc_0156	1.9	0.9
15	Oant_3901	Oant_3900	1.9	0
16	EFER_1531	EFER_1532	2.6	0
17	Csal_2478	Csal_2477	1.9	0.4
18	Apre_1382	Apre_1381	1.3	0.4
19	TTHA_0767	TTHA_0768	4.5	1.7
20	SPO_1772	SPO_1771	3.1	0.4
**Mean % (std. dev.)**			2.1 (0.8)	0.6 (0.5)
**Other transporters for comparison**				
SSS family transporters*, mean % (std. dev.)			1.9 (0.6)	
			scaffold domain	transport domain
DASS family transporters†, mean % (std. dev.)			2.9 (1.2)	1.9 (0.7)

* The InterPro Accession number and gene name (in brackets) for 20 SSS family transporters used for this analysis are: A1AIQ8 (Ecok1_40540), A5IU69 (SaurJH9_195), O07556 (BSU10450), P10502 (STM1125), P16256 (JW3226), P31640, (H16_A2524), P39599 (BSU38240), Q1M7A2 (pRL100404), Q5E733 (VF_0668), Q8NS49 (Cgl0833), A0A009FBY7 (J504_1006), A0A011PZ93 (AW10_00735), A0A015SW20 (M124_1754), A0A017H4T8 (FNF_04561), A0A022L5V2 (D514_0116490), A0A023WN64 (UIB01_01730, A0A024P8B4 (BN983_03685), A0A059FF12 (HJA_07247), A0A059L5U7 (V466_08980) and A0A061PSC2 (JCM19052_352).

† The InterPro Accession number and gene name (in brackets) for 20 DASS family transporters used for this analysis are: Q9KNE0 (VcINDY, VC _A0025), Q5FKK5 (LaINDY, LBA0912), A0A009ER92 (J504_1984), A0A023X051 (BradSPS_0117), A0A024Q941 (BN990_01249), A0A060Q0S5 (NY40_0760), A0A066UIN9 (MBO_00860), A0A075P0L2 (DEB45_13245), A0A076LN52 (ETEE_0663), A0A084ELG6 (HQ939_02825), A0A085Z7Y1 (IX39_07855), A0A087IKG9 (D8T54_17780), A0A096A7S8 (HMPREF1650_06185), A0A096E806 (FZC30_07030), A0A098B799 (DPCES_3845), A0A0A1MEE1 (BN997_01275), A0A0A2WY60 (CF557_10760), A0A0A6YSF4 (HMPREF9074_09352), A0A0A7EGC3 (OM33_11175) and A0A0A8HWC9 (UPTC3659_1361).

Supplementary Table 3 | Data collection and refinement statistics (molecular replacement).

	SiaP-Neu5Ac*
**Data collection**	
Space group	$P2_12_12_1$
Cell dimensions	
a, *b*, *c* (Å)	58.6, 87.3, 129.4
α, β, γ (°)	90, 90, 90
Resolution (Å)	43.66-1.04 (1.077-1.04)
R_{merge}	0.073 (1.153)
$I / \sigma I$	9.8 (1.3)
Completeness (%)	99.6 (93.0)
Redundancy	6.5 (5.7)
**Refinement**	
Resolution (Å)	1.04
No. reflections	316,707 (31,310)
R_{work} / R_{free}	0.1237 / 0.1386
No. atoms	
Protein	5379
Ligand/ion	88
Water	799
B-factors (Å^2)	
Protein	12.7
Ligand/ion	12.5
Water	26.4
R.m.s. deviations	
Bond lengths (Å)	0.009
Bond angles (°)	1.08
PDB	7t3e

The data were collected from a single crystal.
*Values in parentheses are for highest-resolution shell.

Supplementary Table 4 | Interfacial/coevolved residue predictions for SiaQ:SiaM.

Prediction method*	Rank[†]	Predicted probability	Residue pairs		Cβ-Cβ distance[‡] in structure (Å)
			SiaM	SiaQ	
RaptorX complex-contact	1	0.56	Glu-237	Ile-29	5.5
	2	0.54	Ile-240	Leu-25	4.7
	3	0.54	Ile-240	Ile-29	6.3
	4	0.54	Lys-232	Trp-33	8.1
	5	0.54	Ala-42	Tyr-103	4.4
	30	0.49	Gln-267	Val-15	6.8
	31	0.49	Gly-15	Lys-69	5.4
	32	0.49	Ser-243	Leu-22	7.3
	33	0.49	Val-18	Val-68	5.8
	34	0.49	Val-270	Met-53	6.8
GREMLIN	1	1	Leu-244	Thr-26	6.3
	2	1	Ile-23	Thr-96	5.6
	3	1	Asp-50	Arg-47	6.4
	4	1	Lys-232	Trp-33	8.1
	5	1	Thr-271	Lys-64	6.8
	30	0.91	Met-45	Met-100	7.9
	31	0.9	Leu-49	Leu-51	5.5
	32	0.89	Ile-240	Thr-26	6.6
	33	0.89	Ala-277	Phe-50	8.8
	34	0.89	Met-45	Ile-55	9.8

* Each prediction method was run with default settings.

[†] The residue pairs are ranked according to each method's estimate of the probability that a residue pair is in contact. The top five residue pairs are displayed, alongside the residue pairs ranking 30-34 with a lower predicted coupling probability, arbitrarily chosen to show the extent of useful information provided by these analyses. We note that the probabilities are calculated differently between methods, though we have chosen to display them here so they can be compared to the interfacial predictions for SiaM:SiaP, below.

[‡] Cβ-Cβ distance was analysed (Cα-Cβ for residue pairs containing glycine) as residue pairs are typically defined to be in contact if their Cβ-Cβ distance is less than 8 Å.

Supplementary Table 5 | Interfacial/coevolved residue predictions for SiaM:SiaP and SiaQ:SiaP.

Prediction method*	Rank†	Predicted probability	Residue pairs		Residue location (SiaM)	Residue location (SiaP)	Location colour (in Fig. 3a)
			**SiaM**	**SiaP**			
RaptorX complex-contact	1	0.49	Ser-164	Glu-197	L5b-6	η5	Orange (A)
	2	0.49	Thr-234	Ala-179	L7-8	Lα5-β7	Blue (C)
	3	0.49	Pro-235	Leu-173	Tm8	α5	Blue (C)
	4	0.48	Thr-234	Ala-174	L7-8	α5	Blue (C)
	5	0.48	Ala-291	Gln-44	Tm9a	Lβ2-α2	Blue (C)
GREMLIN	1	1	Gly-166	Tyr-172	Tm6	α5	Orange (A)
	2	1	Gln-294	Leu-38	L9a-9b	β2	Blue (B)
	3	0.97	Ser-164	Glu-197	L5b-6	η5	Orange (A)
	4	0.97	Gly-47	Ala-50	Tm3a	α2	Purple (D)
	5	0.86	Pro-235	Leu-173	Tm8	α5	Blue (C)
**Prediction method***	**Rank†**	**Predicted probability**	**Residue pairs**		**Residue location (SiaQ)**	**Residue location (SiaP)**	**Location colour (in Fig. 3a)**
			**SiaQ**	**SiaP**			
RaptorX complex-contact	1	0.49	Thr-118	Pro-165	L3-4	β6	Green (E)
	2	0.49	Ile-117	Pro-165	L3-4	β6	Green (E)
	3	0.48	Pro-38	Ser-162	L1-2	β6	Green (F)
	4	0.47	Thr-118	Ala-149	L3-4	Lβ5-α4	Green (E)
	5	0.45	Leu-40	Pro-163	L1-2	β6	Green (F)
GREMLIN	1	1	Pro-38	Ser-162	L1-2	β6	Green (F)
	2	0.63	Arg-110	Met-57	Tm3	α2-β3	Green (E)
	3	0.59	Arg-110	Ser-56	Tm3	α2	Green (E)
	4	0.55	Leu-40	Pro-163	L1-2	β6	Green (F)
	5	0.47	Arg-110	Asp-219	Tm3	α8	Green (E)

Supplementary Table 6 | SiaPQM mutants tested.

	Analysed with the transport assay	Unable to be purified
**SiaP**	R49A, D59A, K143E, N148D, E170A	D181A, D181K, F195-E197A
**SiaQ**	D36A	
**SiaM**	D50A, S106A/S108A, K232A, R292A, E329A, T369A	

Supplementary Table 7 | Protein sequences of SiaQM and SiaP expressed and purified in this study.

Protein	Protein sequence
SiaQ<br>https://www.uniprot.org/uniprot/Q6LPW0	MFRKIIDNIEEIITVPLMIALLCILTWQISSRWLFDSPSLWSEELARV<br>LFLHMAIIGGAIAIKKDDHVKITFFSDKLPRNFRYSLLFALELLVLIT<br>IVAMIYYGYAHVQRTAFFELITLGISSSWMTYALPVGGCFMLVRQ<br>CQKLYFVLIDWRINENKNTSHLTACDINE
SiaM<br>https://www.uniprot.org/uniprot/Q6LPW1	MFTSIVGWLGLLFAGMPVGFSLIFVGLAFLVLTESTGINFAAQQMI<br>GGLDNFTLLAVPFFVLTGHLMNSAGITERIFNFAKAMVGHITGSLG<br>HVNILASLLFSGMSGSALADAGGLGQLEIKSMRDAKYDDDFAGG<br>LTAASCIIGPLVPPSIPLVIYGVVSNTSIGALFLAGAIPGLLCCIALCI<br>MTYFIAKKRGYMTLPRASRKERLIAFRDAFLSLLTPFIIIGGIFSGKF<br>TPTEAAIISSLYALFLGTVVYKSLTMDKFIKLVQETVTTTSVVALM<br>VMGVTVFGWIVAREQLPQQLAELFLSISDNPLILLLLLINLLLLFLGT<br>FIESLALLLLLVPFLVPVATSVGIDPVHFGVMAILNLMIGILTPPMG<br>MALYVVSKVGNIPFHVLTRGVLPLLVPLFIVLGLIIVFPQITLFLPQL<br>VLGYGL
SiaP<br>https://www.uniprot.org/uniprot/Q6LPV9<br>Underline denotes periplasmic signal peptide	MNNINKITLSLLALALTTTAVQAETILKMGLQANVGSVEYDSAKIL<br>SDKISELSDGEMKLLLYPGAQLGDDRAMLQQLSMGDLDITFAEFG<br>RMGLWIPRAEAVMLPYVVKNYAHIQRIFNSKFGQGVREEMLTNF<br>NWRALDTWYNGTRQTSSNRPLNTISDFEGLKLRVPNAKANLAFA<br>KYAGASPTPMVFSEVYLALQTNAVDGQENPLPTFNTMKFYEVQP<br>NLAMTNHIVNDQMVLISEDRWQSLSKDQQATITEAVSVAGKRHT<br>NFVNNQEKELITFFKAEGVNITYPDLAPFREAMLPIYKDFDKKIGK<br>QLVEELSDI
Megabody (Mb^{c7HopQ}_{Nb07})<br>https://www.uniprot.org/uniprot/B5Z8H1.<br>X=Nb07 β-strand 1 residues 1-13,<br>X=HopQ C-terminal residues 227-446,<br>X=HopQ N-terminal residues 53-221,<br>X=Nb07 residues 14-122	QVQLQESGGGLVQTKTTTSVIDTTNDAQNLLTQAQTIVNTLKDYC<br>PILIAKSSSSNGGTNNANTPSWQTAGGGKNSCATFGAEFSAASDMI<br>NNAQKIVQETQQLSANQPKNITQPHNLNLNSPSSLTALAQKMLKN<br>AQSQAEILKLANQVESDFNKLSSGHLKDYIGKCDASAISSANMTM<br>QNQKNNWGNGCAGVEETQSLLKTSAADFNNQTPQINQAQNLAN<br>TLIQELGNNDTYEQLSRLLTNDNGTNSKTSAQAINQAVNNLNERA<br>KTLAGGTTNSPAYQATLLALRSVLGLWNSMGYAVICGGYTKSPG<br>ENNQKDFHYTDENGNGTTINCGGSTNSNGTHSYNGTNTLKADKN<br>VSLSIEQYEKIHEAYQILSKALKQAGLAPLNSKGEKLEAHVTTSKY<br>AGGSLRLSCAASGNIFDRGYMGWYRQAPGKERELVAGISYGGST<br>YYADSVKGRFTISRDNAKNTVYLQMNSLKPEDTAVYYCAAYPLY<br>DDPYYYWGQGTQVTVSSLE

References

1. Brautigam, C. A. Calculations and publication-quality illustrations for analytical ultracentrifugation data. *Methods Enzymol.* **562,** 109–133 (2015).

2. Zivanov, J., Nakane, T., Forsberg, B. O., Kimanius, D., Hagen, W. J., Lindahl, E. & Scheres, S. H. New tools for automated high-resolution cryo-EM structure determination in RELION-3. *eLife* **7,** 163 (2018).

3. Pettersen, E. F., Goddard, T. D., Huang, C. C., Meng, E. C., Couch, G. S., Croll, T. I., Morris, J. H. & Ferrin, T. E. UCSF ChimeraX: Structure visualization for researchers, educators, and developers. *Protein Sci.* **30,** 70–82 (2021).

4. Stamm, M., Staritzbichler, R., Khafizov, K. & Forrest, L. R. AlignMe—a membrane protein sequence alignment web server. *Nucleic Acids Res.* **42,** W246–W251 (2014).

5. Waterhouse, A. M., Procter, J. B., Martin, D. M. A., Clamp, M. & Barton, G. J. Jalview Version 2—a multiple sequence alignment editor and analysis workbench. *Bioinformatics* **25,** 1189–1191 (2009).

6. Sauer, D. B., Trebesch, N., Marden, J. J., Cocco, N., Song, J., Koide, A., Koide, S., Tajkhorshid, E. & Wang, D.-N. Structural basis for the reaction cycle of DASS dicarboxylate transporters. *eLife* **9,** e61350 (2020).

7. Lomize, A. L., Pogozheva, I. D., Lomize, M. A. & Mosberg, H. I. Positioning of proteins in membranes: A computational approach. *Protein Sci.* **15,** 1318–1333 (2006).

8. Liebschner, D., Afonine, P. V., Baker, M. L., Bunkóczi, G., Chen, V. B., Croll, T. I., Hintze, B., Hung, L.-W., Jain, S., McCoy, A. J., Moriarty, N. W., Oeffner, R. D., Poon, B. K., Prisant, M. G., Read, R. J., Richardson, J. S., Richardson, D. C., Sammito, M. D., Sobolev, O. V., Stockwell, D. H., Terwilliger, T. C., Urzhumtsev, A. G., Videau, L. L., Williams, C. J. & Adams, P. D. Macromolecular structure determination using X-rays, neutrons and electrons: recent developments in Phenix. *Acta Crystallogr. D.* **75,** 861–877 (2019).

9. Laskowski, R. A. & Swindells, M. B. LigPlot+: Multiple Ligand–Protein Interaction Diagrams for Drug Discovery. *J. Chem. Inf. Model* **51,** 2778–2786 (2011).

10. Webb, B. & Sali, A. Comparative protein structure modeling using MODELLER. *Curr. Protoc. Bioinform.* **54,** 5.6.1-5.6.37 (2016).

11. Brautigam, C. A., Zhao, H., Vargas, C., Keller, S. & Schuck, P. Integration and global analysis of isothermal titration calorimetry data for studying macromolecular interactions. *Nat. Protoc.* **11,** 882–894 (2016).

12. Mancusso, R., Gregorio, G. G., Liu, Q. & Wang, D.-N. Structure and mechanism of a bacterial sodium-dependent dicarboxylate transporter. *Nature* **491,** 622–626 (2012).

13. Bolla, J. R., Su, C.-C., Delmar, J. A., Radhakrishnan, A., Kumar, N., Chou, T.-H., Long, F., Rajashankar, K. R. & Yu, E. W. Crystal structure of the *Alcanivorax borkumensis* YdaH transporter reveals an unusual topology. *Nat. Commun.* **6,** 6874–6874 (2015).

14. Prakash, S., Cooper, G., Singhi, S. & Saier, M. H. The ion transporter superfamily. *Biochim. Biophys. Acta Biomembr.* **1618,** 79–92 (2003).

15. Mulligan, C., Geertsma, E. R., Severi, E., Kelly, D. J., Poolman, B. & Thomas, G. H. The substrate-binding protein imposes directionality on an electrochemical sodium gradient-driven TRAP transporter. *Proc. National Acad. Sci. USA.* **106,** 1778–1783 (2009).
