# Supplementary material for: Structure and mechanism of a tripartite ATP-independent periplasmic TRAP transporter
Source: Nat Commun. 2023 Feb 27;14:1120. doi: 10.1038/s41467-023-36590-1 (PMC9971032; doi:10.1038/s41467-023-36590-1)
Supplement: Supplementary file 3 — Description of Additional Supplementary Files [file 41467_2023_36590_MOESM3_ESM.pdf]

File name: Supplementary Data 1

Description: PpSiaPQM tripartite model Rosetta relaxed

File name: Supplementary Data 2

Description: PpSiaPQM tripartite model with Modeller outward facing SiaM
