# Supplementary material for: Structure and mechanism of a tripartite ATP-independent periplasmic TRAP transporter
Source: Nat Commun. 2023 Feb 27;14:1120. doi: 10.1038/s41467-023-36590-1 (PMC9971032; doi:10.1038/s41467-023-36590-1)
Supplement: Supplementary file 6 — Reporting Summary [file 41467_2023_36590_MOESM6_ESM.pdf]

nature portfolio

Corresponding author(s): Prof. Renwick Dobson

Last updated by author(s): Oct 26, 2022

# Reporting Summary

Nature Portfolio wishes to improve the reproducibility of the work that we publish. This form provides structure for consistency and transparency in reporting. For further information on Nature Portfolio policies, see our Editorial Policies and the Editorial Policy Checklist.

## Statistics

For all statistical analyses, confirm that the following items are present in the figure legend, table legend, main text, or Methods section.

| n/a | Confirmed | |
|---|---|---|
| ☐ | ☒ | The exact sample size (*n*) for each experimental group/condition, given as a discrete number and unit of measurement |
| ☐ | ☒ | A statement on whether measurements were taken from distinct samples or whether the same sample was measured repeatedly |
| ☐ | ☒ | The statistical test(s) used AND whether they are one- or two-sided<br>*Only common tests should be described solely by name; describe more complex techniques in the Methods section.* |
| ☒ | ☐ | A description of all covariates tested |
| ☒ | ☐ | A description of any assumptions or corrections, such as tests of normality and adjustment for multiple comparisons |
| ☐ | ☒ | A full description of the statistical parameters including central tendency (e.g. means) or other basic estimates (e.g. regression coefficient) AND variation (e.g. standard deviation) or associated estimates of uncertainty (e.g. confidence intervals) |
| ☐ | ☒ | For null hypothesis testing, the test statistic (e.g. *F*, *t*, *r*) with confidence intervals, effect sizes, degrees of freedom and *P* value noted<br>*Give P values as exact values whenever suitable.* |
| ☒ | ☐ | For Bayesian analysis, information on the choice of priors and Markov chain Monte Carlo settings |
| ☒ | ☐ | For hierarchical and complex designs, identification of the appropriate level for tests and full reporting of outcomes |
| ☒ | ☐ | Estimates of effect sizes (e.g. Cohen's *d*, Pearson's *r*), indicating how they were calculated |

*Our web collection on statistics for biologists contains articles on many of the points above.*

## Software and code

Policy information about availability of computer code

| | |
|---|---|
| Data collection | ProteomeLab XL-I and Optima AUC was used to acquire analytical ultracentrifugation data.<br>EPU software package (Thermo Fisher Scientific, ver. 2.6) was used for all automated cyro-electron microscopy data collection and autofocus<br>AlphaFold2 was used with Colab notebook. |
| Data analysis | SEDFIT (ver. 16.2c) and UltraScan (ver. 4.0, release 2843) was used to process all analytical ultracentrifugation data.<br>GraphPad Prism (ver. 8.3.0) was used for the nonlinear regression (curve fit).<br>The CCP4 programme suite (ver. 7.1), Phenix software package (ver. 1.17.1-3660), RELION (ver. 3.0), cryoSPARC (v.3.2.0), and COOT (ver. 0.9.5) were used for all data processing and structure refinement for our Crystallography and Cryo-EM experiments.<br>The ATSAS software package (ver. 3.0) was used to analyse all SAXS data.<br>The SiaQM:SiaP complex was predicted using AlphaFold33, with experimentally determined structures then aligned to this complex. Rosetta fast relax was then used to improve the sidechain packing of the complex<br>Thermal shift data was analysed using Protein Thermal Shift Software v1.4 (Applied Biosystems) |

For manuscripts utilizing custom algorithms or software that are central to the research but not yet described in published literature, software must be made available to editors and reviewers. We strongly encourage code deposition in a community repository (e.g. GitHub). See the Nature Portfolio guidelines for submitting code & software for further information.

## Data

Policy information about availability of data

All manuscripts must include a data availability statement. This statement should provide the following information, where applicable:

- Accession codes, unique identifiers, or web links for publicly available datasets
- A description of any restrictions on data availability
- For clinical datasets or third party data, please ensure that the statement adheres to our policy

The data generated or analysed in this study are available from the corresponding author (R.C.J.D.) upon reasonable request. The crystal structures and data are available from the PDB (code 7t3e) and the cryo-EM structures and data are available from the PDB (codes 7qha and 8b01) and the EMDB (codes EMD-13968 and EMD-15775). The coordinates for the SiaPQM complex model predicted by AlphaFold are available from the corresponding author on reasonable request.

## Human research participants

Policy information about studies involving human research participants and Sex and Gender in Research.

| | |
|---|---|
| Reporting on sex and gender | N/A |
| Population characteristics | N/A |
| Recruitment | N/A |
| Ethics oversight | N/A |

Note that full information on the approval of the study protocol must also be provided in the manuscript.

# Field-specific reporting

Please select the one below that is the best fit for your research. If you are not sure, read the appropriate sections before making your selection.

☒ Life sciences ☐ Behavioural & social sciences ☐ Ecological, evolutionary & environmental sciences

For a reference copy of the document with all sections, see nature.com/documents/nr-reporting-summary-flat.pdf

# Life sciences study design

All studies must disclose on these points even when the disclosure is negative.

| | |
|---|---|
| Sample size | The sample size for the functional studies was a judgment between expense (time and cost of radio labeled sialic acid) and minimizing the error associated with each measurement. |
| Data exclusions | None |
| Replication | The number of replicates are indicated in the figure legends. |
| Randomization | N/A. Randomization is used in animal/clinical studies, but does not apply for structural biology experiments (cryoEM or crystallography). Note that the R-free set for the crystal structure was randomly chosen. |
| Blinding | N/A. The study does not include animal or clinical studies. |

# Reporting for specific materials, systems and methods

We require information from authors about some types of materials, experimental systems and methods used in many studies. Here, indicate whether each material, system or method listed is relevant to your study. If you are not sure if a list item applies to your research, read the appropriate section before selecting a response.

## Materials & experimental systems

| n/a | Involved in the study |
| --- | --- |
| ☒ | ☐ Antibodies |
| ☒ | ☐ Eukaryotic cell lines |
| ☒ | ☐ Palaeontology and archaeology |
| ☒ | ☐ Animals and other organisms |
| ☒ | ☐ Clinical data |
| ☒ | ☐ Dual use research of concern |

## Methods

| n/a | Involved in the study |
| --- | --- |
| ☒ | ☐ ChIP-seq |
| ☒ | ☐ Flow cytometry |
| ☒ | ☐ MRI-based neuroimaging |
